# Neural correlates of moral judgments in first- and third-person perspectives: implications for neuroethics and beyond

**DOI:** 10.1186/1471-2202-15-39

**Published:** 2014-04-01

**Authors:** Mihai Avram, Kristina Hennig-Fast, Yan Bao, Ernst Pöppel, Maximilian Reiser, Janusch Blautzik, James Giordano, Evgeny Gutyrchik

**Affiliations:** 1 Institute of Medical Psychology, Ludwig-Maximilians-Universität, Munich, Germany; 2 Human Science Center, Ludwig-Maximilians-Universität, Munich, Germany; 3 Clinic of Psychiatry and Psychotherapy, Ludwig-Maximilians-Universität, Munich, Germany; 4 Institute of Clinical Radiology, Ludwig-Maximilians-Universität, Munich, Germany; 5 Department of Psychology and Key Laboratory of Machine Perception (MoE), Peking University, Beijing, People's Republic of China; 6 Parmenides Center for Art and Science, Munich, Germany; 7 Neuroethics Studies Program, Pellegrino Center for Clinical Bioethics, Georgetown University Medical Center, Washington, DC, USA; 8 Inter-disciplinary Program in Neuroscience, Georgetown University Medical Center, Washington, DC, USA

**Keywords:** fMRI, Moral judgment, Perspective, "actor-observer bias", Anterior medial prefrontal cortex, Precuneus, Hippocampus, Theory of mind, Neuroethics

## Abstract

**Background:**

There appears to be an inconsistency in experimental paradigms used in fMRI research on moral judgments. As stimuli, moral dilemmas or moral statements/ pictures that induce emotional reactions are usually employed; a main difference between these stimuli is the perspective of the participants reflecting first-person (moral dilemmas) or third-person perspective (moral reactions). The present study employed functional magnetic resonance imaging (fMRI) in order to investigate the neural correlates of moral judgments in either first- or third-person perspective.

**Results:**

Our results indicate that different neural mechanisms appear to be involved in these perspectives. Although conjunction analysis revealed common activation in the anterior medial prefrontal cortex, third person-perspective elicited unique activations in hippocampus and visual cortex. The common activation can be explained by the role the anterior medial prefrontal cortex may play in integrating different information types and also by its involvement in theory of mind. Our results also indicate that the so-called "actor-observer bias" affects moral evaluation in the third-person perspective, possibly due to the involvement of the hippocampus. We suggest two possible ways in which the hippocampus may support the process of moral judgment: by the engagement of episodic memory and its role in understanding the behaviors and emotions of others.

**Conclusion:**

We posit that these findings demonstrate that first or third person perspectives in moral cognition involve distinct neural processes, that are important to different aspects of moral judgments. These  results are important to a deepened understanding of neural correlates of moral cognition—the so-called “first tradition” of neuroethics, with the caveat that any results must be interpreted and employed with prudence, so as to heed neuroethics “second tradition” that sustains the pragmatic evaluation of outcomes, capabilities and limitations of neuroscientific techniques and technologies.

## Background

Studies of moral decision-making have been the focus of philosophy, psychology, and more recently, the brain sciences. Examination of the ways that humans (and perhaps other organisms) engage intent, memory, emotion, and reasoning processes relevant to their execution and constraint of conduct toward others, acquisition and use of various resources, survival, and flourishing have become the emphases of sub-disciplines of the cognitive neurosciences, such as neuroeconomics and more specifically, neuroethics. Developing from the older fields of moral philosophy and moral psychology, neuroethics obtains two primary orientations (or so-called “traditions”). The first can be somewhat colloquially described as “..the neuroscience of ethics” [1]. Rather, we offer that a more apt definition of this branch of neuroethics would be: studies of the putative neural substrates and mechanisms involved in proto-moral and moral cognition and behaviors [2-5]. The second “tradition” addresses the ethico-legal and social issues fostered by the use of neuroscience and neurotechnologies in research, medical practice, or public life.

In this latter regard, particular interest has centered upon the use of neuroimaging techniques and technologies to depict, and define neural bases of moral decision-making, if not “morality”, writ-large–as constituent to ongoing criticism of neuroimaging, in general [6]. Still, by recognizing and compensating inherent technical and conceptual limitations [7] iterative progress in neuroimaging technology and method have yielded improvement in outcomes, which sustain this approach as both valid and valuable to elucidating the relative activity of various neural networks in certain types of cognitive tasks and behaviors, including those involved in moral judgments and behaviors - with certain caveats noted and acknowledged [8,9].

Such studies have revealed the complexity of these types of decisions. In the main, focus has shifted from defining moral judgments as purely cognitive processes (i.e. - reason) to revealing more emotion-based processes, and recent results suggest the involvement of both processes in those decisions that are (both subjectively and objectively evaluated as being) morally sensitive and/or responsive [10-15]. What has also become clear is that moral decisions are not uniformly processed by a particular locus, region or network [16,17], but rather are more widely distributed in and across neural fields that are involved in memory, reward, reinforcement, and punishment, rationalization, interoception (e.g.- provocation of and response to various emotions, self-referentiality, etc.), and behavior. For example, Young and Dungan [18] suggest that such brain areas include the medial prefrontal cortex (MPFC) – involved in emotional processing; posterior cingulate cortex (PCC) and precuneus – both involved in self-referential processing, the temporo-parietal junction (TPJ) and/or somewhat larger fields of Brodmann’s area 39 – that are involved in aspects of social processing and/ or theory of mind (ToM).

As well, it is likely that different patterns of neural network activation may be involved in particular types of moral decisions, based upon the nature of the evocative stimuli, situations, and relative involvement of the subject. In this light, a methodological question has recently been raised regarding the viability of the rational and emotional/ intuitionist theories of moral cognition and judgments [19]. These research approaches to moral judgment use different experimental stimuli: “rationalist” protocols use moral dilemmas to study moral judgments, while “emotionalist” protocols employ emotionally-laden statements or pictures to assess what appear to be moral reactions. Is it possible that these approaches elicit distinct processes of moral cognition and lead to different results? Monin and colleagues [19] argue that the focus of reasoning in moral dilemmas is on the decision-making process - a conflict between two moral constructs and/or principles, whereas moral reactions reflect subjects’ emotional responses to particular stimuli and situations that have moral relevance. Of note is that moral dilemma protocols are typically presented in a first person perspective (1PP), while moral reaction protocols are characteristically presented in a third-person perspective (3PP). Thus, we question whether the perspective of the subject(s) toward the moral stimuli is sufficient to evoke differing effects, and elicit distinct patterns of neural network activity.

We opine that using stimuli presented in either 1- or 3PP may elucidate a number of potentially interactive variables that may shed new light on studies of neural mechanisms and processes of moral cognition. To wit, it has been shown that different patterns of neural activity were observed for stimuli presented in either 1- or 3-PP in *non-moral visuospatial tasks*[20]. During the 1-PP situation, neural activity was increased in the medial prefrontal cortex (MPFC), posterior cingulate cortex (PCC), and temporoparietal junction (TPJ) bilaterally, whereas in the 3-PP situation, neural activity was increased in the medial superior parietal and right premotor cortex.

Furthermore, differences have also been found in social non-moral tasks (which appear to reflect theory of mind, ToM), although these results are somewhat less clear. In a study on the influence of the person's perspective on ToM, 1- and 3-PP-type sentences elicited different patterns of neural activation: 1PP-based stimuli yielded greater activation in the caudate nucleus, while 3PP-based stimuli evoked increased neural activity in the dorsolateral prefrontal cortex (DLPFC). The authors related activity in the caudate nucleus to self-focal cognition, and DLPFC-activity to ToM. Other studies report stronger 3PP activation in the TPJ and dorsal MPFC [21-24] which are regarded as parts of the ToM network.

On the other hand, many of these studies have reported greater activation for the 1PP compared to 3PP in the MPFC and PCC/ precuneus. Ochsner and colleagues compared neural processes involved in inferences about one's own and others emotional states. Concomitant activation was demonstrated in the MPFC, left inferior PFC, PCC/ precuneus and STS/ TPJ [25]. This appeared to reflect recruitment of specific sub-regions in the MPFC, and additional activation in the medial temporal cortex for processing self-emotionality, while the lateral PFC and medial occipital activation appeared to be involved in processing emotional inferences of/about others. We posit that these results suggest that "self-judgments" seem to activate more medial networks, while judgments about others appear to engage more lateral networks. As well, components of both networks have some degree of overlap.

Social psychological studies have repeatedly shown that negative situations elicit a tendency to attribute one's own actions (1PP) to external causes, while attributing other people's (3PP) behaviors to internal causes, a phenomenon referred to as the "actor- observer bias" [26,27]. This may affect results in studies of moral decision-making, given that many such studies have employed negative situations as stimuli [28]. Nadelhoffer and Feltz [27] conducted a behavioral study of the actor-observer bias using a version of Philippa Foot’s [29] iconic "trolley problem" as the moral dilemma stimulus, viz.- a trolley is running out of control toward five people who are on the track and unaware of the looming danger. You have the opportunity to save these five people by throwing a switch and sending the trolley down a different track. However, if you do this, you will then kill one individual who is on the second track (for overview, see also Thomson [30] and for discussion of relevance to neural bases of moral decision-making, see Green [31]). The dilemma was presented either in a 1PP (i.e. - the subject was the actor, actively engaged in throwing the switch to divert the trolley), or in a 3PP (i.e. - the subject was a passive observer who could tell an actor to throw the switch). In the actor condition, 65% of the participants found the action (throwing the switch) to be permissible, whereas 90% of the participants in the observer condition found the action to be morally acceptable. These results imply different psychological processes involved in the two perspectives.

Thus, differential activation of distinct neural networks in response to 1PP- or 3PP-based stimuli is expected. Based on previous studies activation in the medial parts of the default mode network can be anticipated for the 1PP, and more lateral activation (e.g. DLPFC, TPJ) can be expected for the 3PP. However, since common activation for both perspectives has been found in several studies, and the default mode and ToM networks overlap in several regions, shared activation may also be expected. MPFC and PCC/ precuneus seem to be common denominators for the perspectives. Theoretically, the observer condition (3PP) of the "actor- observer bias" would tend to involve attribution of behaviors to internal causes, thus there is an attempt to understand the mind (i.e. - mental processes, in this case, the perceived “morality”) of the "actor". Indeed, ToM has been linked to moral judgments, and may be seen as important to moral evaluations of the actions of others [18].

As well, given that (a) most decisions, inclusive of potentially moral judgments involve some degree of Bayesian processing [32,33]; (b) such processing involves recollection of circumstance, effect and potential consequences in orientation to self, others and situations [2,5,34], and (c) learning and memory have been shown to play significant roles in these processes [35,36], it is likely that neural substrates of memory (e.g.- septo-hippocampal networks) would be involved [37,38]. Studies have fortified this speculation by demonstrating hippocampal activation in tasks involving perception of the emotions and actions of others [39,40]. Accordingly, we posit that hippocampal activation (for the 3PP-, as well as perhaps 1PP-situations) is to be expected. In sum, we hypothesize that the perspective of the subject (i.e.- as either actor (1PP), or observer (3PP)) will evoke differential activity in distinct neural networks that are putatively involved in the particular cognitive aspects of these orientations to moral judgment(s). To test this hypothesis we employed functional magnetic resonance imaging (fMRI) to compare moral judgments posed in 1- and 3PP-based scenarios.

## Method

### Participants

Sixteen (16) right-handed subjects (9 female, 7 male; mean age 28.25 years) with normal or corrected to normal vision participated in this study. Participants had no reported history of psychiatric or neurological disorder, and were not using psychoactive drugs at the time of the study. The study was conducted in accordance with the Declaration of Helsinki, and approved by the Ethics Committee and Internal Review Board of the Human Science Center of the Ludwig-Maximilians University. Active, written informed consent for participation in the study was obtained from all participants, and subjects received financial compensation for their time.

### Stimulus material

Sixty-nine (69) subjects evaluated 72 moral statements for valence and arousal in a pre-study. Half of the statements were presented in the 1PP ("I am a cruel person because I have aggressive thoughts towards my child"), and half were presented in the 3PP "A person who has aggressive thoughts toward his/ her child is cruel"). To assure valid comparisons, a five point Likert scale was used to rate the stimuli for valence, with scores ranging between −2 (unpleasant) and 2 (pleasant), and arousal, with scores ranging between −2 (agitating) and 2 (calming). Extreme values were excluded on an [−1, 1] interval in order to obviate the strongly emotion- laden stimuli, and to compare similar emotional reactions. Only 8 stimuli remained in each category after the pre-study. In order to ensure valid statistical comparisons of valence and arousal, two paired t-tests were used; there were no statistically significant differences between stimuli presented in 1PP narrative (*M* = −0.82, *SD* = 0.35) and 3PP narrative (*M* = −0.82, *SD* = 0.19), *t* (7) = 0.05, *p* > .05 with respect to valence. There were also no statistically significant differences between stimuli presented in 1PP narrative (*M* = −0.76, *SD* = 0.30) and 3PP narrative (*M* = −0.77, *SD* = 0.22), *t* (7) = 0.04, *p* > .05 with respect to arousal. Another paired *t*-test was used to control for stimulus sentence length. There were no statistically significant differences between stimuli presented in 1PP narrative (*M* = 8.38, *SD* = 3.20) and 3PP narrative (*M* = 10.25, *SD* = 2.71), *t* (7) = 1.34, *p* > .05.

Subjects had to rate the sentences as "right" or "wrong" by relying upon intuition (i.e.- described to them as “a gut-feeling”), and not necessarily their real life experience(s) (e.g. some participants may not have had children), so as to base their answers upon an "as-if” situation (e.g. If I *were* to have aggressive thoughts towards my child - and, indeed, if I had children - would I be a cruel person?).

Although the stimuli were controlled for length, there may have been differences in sentence construction. For example, in the 1PP narrative, "I am a cruel person because I have aggressive thoughts towards my child", it might seem that the 3PP narrative that would have been the best match would be: "John is a cruel person because he has aggressive thoughts towards his child". However, the actor-observer bias appears to be more prominent in cases where the actor is not known - e.g. a stranger [26]. Therefore, we choose a more abstract expression, namely "a person”. Another condition was also used, in which participants were asked to evaluate a non-moral statement based upon their perception of what they believed to be right or wrong (e.g. "There are people who are friendly"). An additional, "scrambled" condition was also used, in which participants had to push a response button when viewing a sentence composed of random letters. This condition was employed to test whether moral judgments activate a similar pattern when compared to scrambled words as in our previous study [14] and is not directly related to this study.

All stimuli were presented twice during the fMRI experiment.

### Procedure

Functional magnetic resonance imaging (fMRI) was used in order to study the 1PP and 3PP types of judgments. A block design was used with 4 conditions (1PP, 3PP, non-moral, and scrambled) and 8 blocks per condition, each block comprising 2 stimuli, presented in white, on a black background. The order of stimuli and blocks was pseudo-randomized. Subjects viewed the stimuli via a mirror attached to the head-coil on a LCD screen behind the scanner. Stimuli were presented for 6000 ms (Presentation, Neurobehavioral Systems, USA), followed by 300 ms displaying a black screen, which in turn was followed by a 1000 ms black screen with a white question mark, in which subjects had to decide whether the statements could be considered right or wrong by pressing a button (Cedrus Lumina response box, Cambridge Research Systems Ltd.). After the two stimuli a black screen was presented for 6000 ms as a break between blocks. This method was used to ensure consistent parameters of cognitive processing in each subject for each presented stimuli. Given these protocols, reaction time analyses were not required.

The study was conducted with a 3T system (Philips ACHIEVA, Germany) at the University Hospital LMU Munich. For anatomical reference, a T1-weighted MPRAGE sequence was performed (TR = 7.4 ms, TE = 3.4 ms, FA = 8°, 301 sagittal slices, FOV = 240 × 256 mm, matrix = 227 × 227, inter-slice gap = 0.6 mm). For BOLD imaging, a T2*-weighted EPI sequence was used (TR = 3000 ms, TE = 35 ms, FA = 90°, 36 axial slices, slice thickness = 3.5 mm, inter-slice gap = 0 mm, ascending acquisition, FOV = 230 × 230 mm, matrix = 76 × 77, in-plane resolution = 3 × 3 mm). In total 229 functional volumes were acquired, 5 being discarded.

### Data processing and analysis

The preprocessing and statistical analyses were performed using SPM8 (Wellcome Department of Cognitive Neurology, London, UK). Motion correction, realignment and spatial normalization were performed in the preprocessing analysis. Smoothing was executed using a Gaussian kernel of 8 mm FWHM. The four experimental conditions were modeled by a boxcar function convolved with a hemodynamic response function. In the first level, several single-tailed t-contrasts have been calculated for each subject, condition versus baseline. The individual contrast images were used for a random effect analysis in a second level. A conjunction analysis was performed to identify positive changes in BOLD signal intensity commonly seen in 1PP and 3PP presentations by using contrast images of each condition compared with the non-moral condition. Only activations are reported. Group activation contrasts (uncorrected < .005) were cluster-level corrected by family wise error (FWE) < .05 with a cluster-size threshold of 50 voxels.

### Region of interest (ROI) analysis

Parameter estimates of signal intensity were extracted from regions of interest (ROIs) for each subject using MARSeille Boîte À Région d’Intérêt software (MarsBaR v0.42; [43] in the aMPFC, precuneus, TPJ, and hippocampus, with ROIs defined as spheres with 10mm radius centered at the peaks of the parametric activation. Anatomical description was accomplished by referring to the Automatic Anatomic Labeling (AAL) [41] atlas from the Wake Forest University (WFU) Pickatlas (Advanced NeuroScience Imaging Research Laboratory, Winston-Salem, North Carolina, USA). Repeated measures analyses of variance with mean beta values for each subject were done to determine whether neural activity within these regions differed between 1- and 3PP moral judgments and the non-moral condition. Gaussian distribution, homogeneity of variance and covariance and sphericity could be assumed (*p* > .05). Corrections for multiple comparisons were done by the Bonferroni procedure. Statistical analyses calculated with SPSS Statistics 16.0 (IBM, USA).

## Results

### Behavioral results

Subjects evaluated the moral statements to be either morally right, or morally wrong.

A chi-square-test revealed a statistically significant difference in yes/ no responses for the two moral conditions, *χ*2 (1) = 28.96, p < 0.01. The participants found 19% of the 1PP and 51% of the 3PP stimuli to be morally right.

### fMRI results

**Figure 1:**
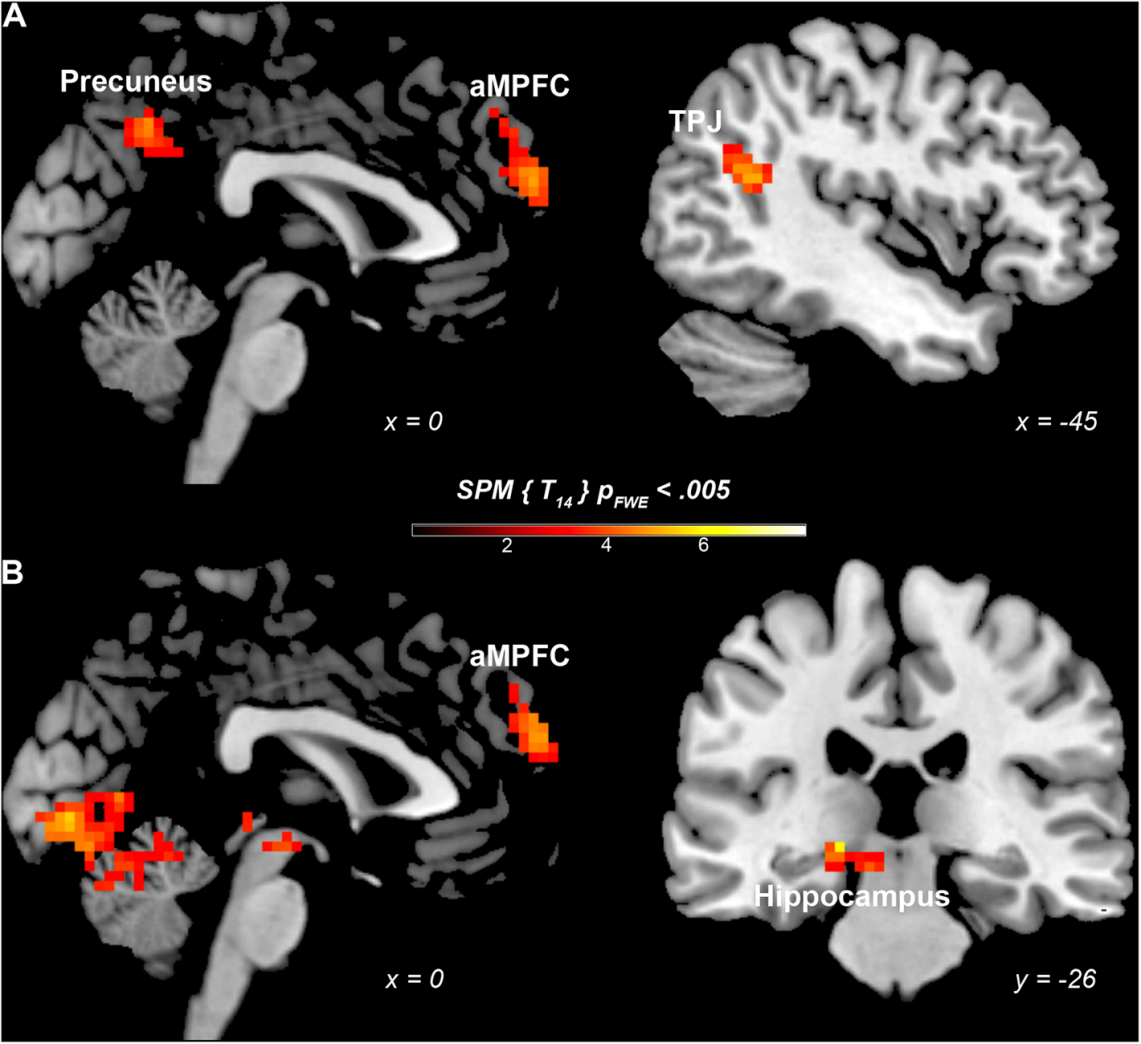
**Neurofunctional correlates of 1- and 3PP moral judgments. (A)** 1PP moral judgments versus NM condition, **(B)** 3PP moral judgments versus NM condition. Anterior Medial Prefrontal Cortex (aMPFC), Temporoparietal Junction (TPJ).

1PP- and 3PP-based judgments were each compared to the non-moral condition (NM). 1PP-based judgments yielded greater activation than NM in the anterior medial prefrontal cortex (aMPFC - BA 10), posterior cingulate cortex (PCC - BA 23) extending in the precuneus (BA 7), and temporoparietal junction (TPJ - BA 39) (Table 1, Figure 1). 3PP-based judgments elicited greater activation in the aMPFC (BA 10), but also in the lingual gyrus (BA 17), middle occipital gyrus (BA 18) and hippocampus (Table 1, Figure 1).

**Table 1:** Relative activation table: 1- and non 3PP moral judgments versus non-moral judgments

	**Left**						**Right**					
**Brain region**	**BA**	**x**	**y**	**z**	**t**	**mm**^**3**^	**BA**	**x**	**y**	**z**	**t**	**mm**^**3**^
***1PP > NM***												
aPFC	10	−6	56	22	5.64	3080	10	12	56	22	3.35	1593
Posterior cingulate cortex	23	−3	−52	31	3.94	378						
Precuneus	7	−3	−58	40	4.98	1431						
Temporoparietal junction	39	−42	−55	19	5.22	675						
***3PP > NM***												
aPFC	10	−15	50	37	4.87	459	10	3	59	28	4.90	1880
Lingual gyrus	18	−33	−85	4	6.69	3726						
Middle occipital gyrus	−22	−25	−8	4.28	378							
Hippocampus	17	6	−82	−5	7.91	4212						

Note. BA – aPFC - anterior prefrontal cortex, Brodmann area, x, y, z – MNI coordinates.

**Figure 2:**
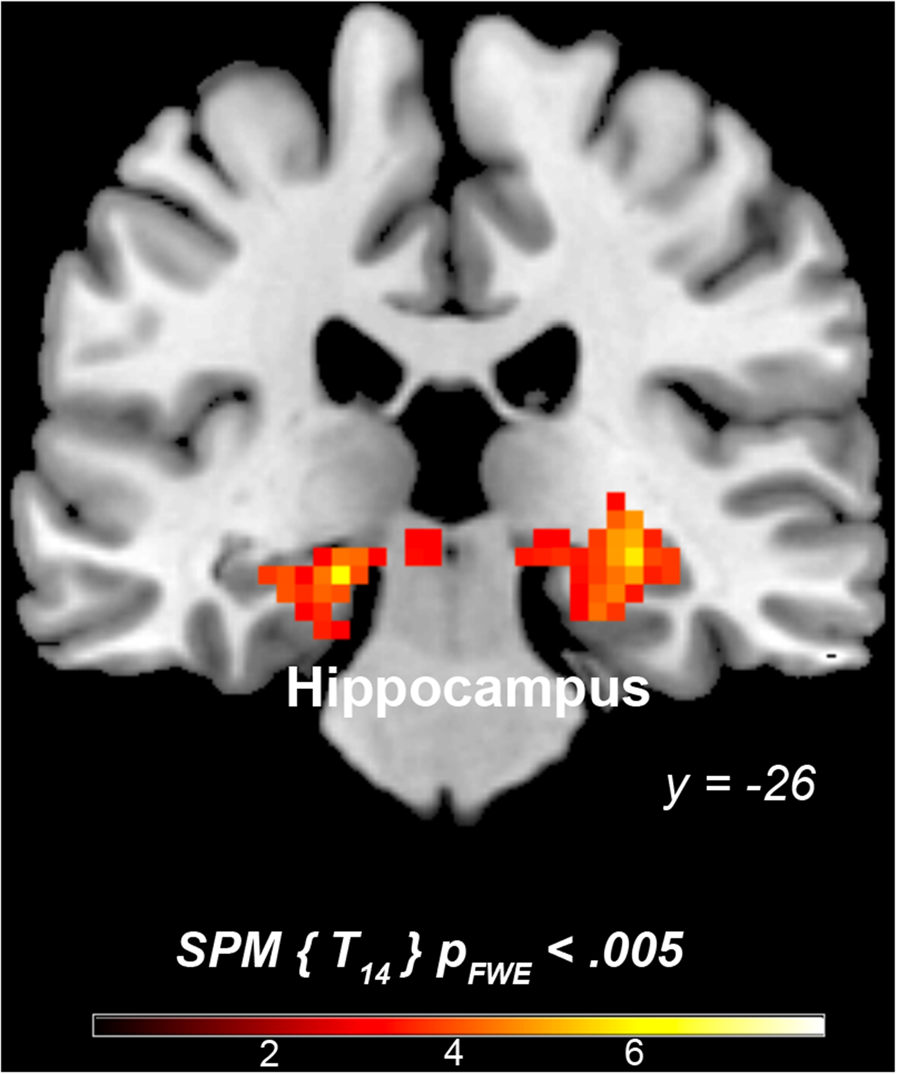
Neurofunctional correlates of 3- vs 1PP moral judgments.

In order to assess overlapping neural activity evoked by the two judgment modalities, a conjunction analysis was used. Common activation for the two judgment modalities (compared to control) was found only in the anterior medial prefrontal cortex *x* = 3, *y* = 59, *z* = 28 (BA 10; cluster size = 3078 mm3, t = 4.93.).Relative activations were generated only by the 3PP > 1PP contrast in: hippocampus bilaterally, and visual cortex - fusiform gyrus (BA 37), middle occipital gyrus (BA 19), and cuneus (BA 18) (Table 2, Figure 2). No activations above threshold were observed in the inversed contrast, 1PP > 3PP.

**Table 2:** Relative activation table: 3PP versus 1PP moral judgments

	**Left**						**Right**					
**Brain region**	**BA**	**x**	**y**	**z**	**t**	**mm**^**3**^	**BA**	**x**	**y**	**z**	**t**	**mm**^**3**^
Hippocampus		−36	−22	−14	4.08	1688		24	−28	−11	5.24	1836
Fusiform gyrus	37	−33	−52	−17	6.05	2889	19	24	−70	−14	4.43	1832
Middle occipital gyrus	19	−30	−85	16	8.25	2584	19	27	−85	19	4.69	2448
Cuneus	18	12	−88	19	4.90	536						

Note. BA – Brodmann area, x, y, z – MNI coordinates.

**Figure 3:**
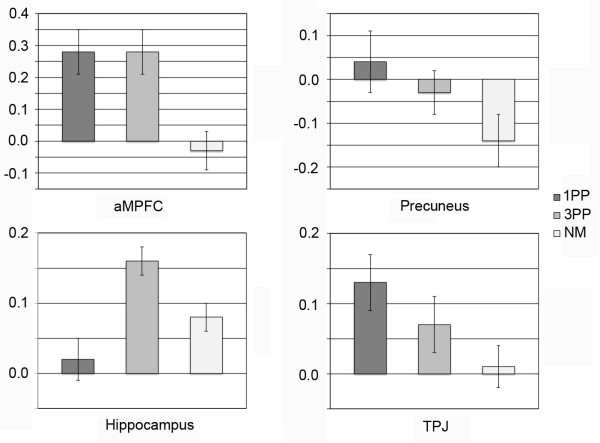
**Region of interest analysis: anterior medial prefrontal cortex (aMPFC), precuneus, hippocampus, and temporoparietal junction (TPJ).** Error bars denote standard error of the mean.

In order to ensure that the effects were related to the 1PP or 3PP moral conditions, and not due to the subtraction of the NM condition, the aMPFC, precuneus, TPJ, and hippocampus were selected for ROI analyses. Overall main effects were observed for all ROIs. For aMPFC (*F*(2, 30) = 13.17, *p* < .001, partial η2 = .468), differences were found between 1PP and NM condition (*p* < .002), and between 3PP and NM conditions (*p* < .006), but no difference was found between the two moral conditions (*p* = 1). For precuneus (*F*(2, 30) = 5.22, *p* < .011, partial η2 = .258) differences were found between 1PP and NM condition (*p* < .038), but none between 3PP and the NM condition (*p* = .057) or between the two moral conditions (*p* = .544). For TPJ (*F*(2, 30) = 7.29, *p* < .003, partial η2 = .327) differences were found between 1PP and NM condition (*p* < .003), and between 3PP and NM conditions (*p* < .032). No difference was found between the moral conditions (*p* = .262). For hippocampus (*F*(2, 30) = 12.46, *p* < .0001, partial η2 = .453) differences were observed between 1PP- and 3PP conditions (*p* < .0001), and between 3PP and NM condition (*p* < .005). However, no difference was found between NM and 1PP conditions (*p* = .316) (Figure 3).

## Discussion

The findings bring to light both common and distinct activations for moral judgments in 1PP and 3PP. A conjunction analysis revealed common activation in the aMPFC for both perspectives. When compared to the non-moral condition, 1PP moral judgments elicited activation in the aMPFC, PCC extending in the precuneus, and TPJ, whereas 3PP moral judgments elicited activation in the aMPFC, hippocampus and visual cortex.

The behavioral results, which revealed that 19% of the stimuli in 1PP- and 51% of the 3PP- stimuli were evaluated as right, seem to concur with Nadelhoffer and Feltz's study [27] showing involvement of the “actor-observer bias”. However, the paucity of imaging research on the “actor-observer bias“ makes it challenging to describe the way in which the neurofunctional correlates of the bias may be contributory to, or form moral judgments.

Even though first and third person perspectives (1PP, 3PP) elicited additional activity (except for aMPFC) in comparison with the non-moral condition (NM), these differences did not withstand the threshold-correction (except for hippocampus and visual cortex) in the direct (3PP- vs.1PP; 1PP vs. 3PP-based comparisons). The findings reveal both common and distinct activations for moral judgments in 1PP and 3PP. A conjunction analysis revealed common activation in the aMPFC for both perspectives. When compared to the non-moral condition, 1PP moral judgments elicited activation in the aMPFC, PCC extending in the precuneus, and TPJ, whereas 3PP moral judgments elicited activation in the aMPFC, hippocampus and visual cortex.

No significant statistical differences in signal activation strength were revealed by the ROI analyses between 1- and 3PP-based presentations in the MPFC, precuneus, and TPJ. The aMPFC has been shown to be involved in the explicit representation of both one’s own mental state, and also the mental states of others [43]. Furthermore, its activity has been consistently demonstrated in social cognition and ToM tasks [42]. Moreover, the aMPFC seems to function in coordination of external and internal stimuli [44].

Theoretically, 1PP presentation should elicit activation in those areas involved in assessing behavior in a given situation. When compared to the non-moral condition, signal activation was elicited in aMPFC, precuneus and right TPJ. Given that in 81% of the cases the subjects evaluated the moral stimuli as wrong; it seems that subjects may have tried to distance themselves from strong emotional stimuli. Koenigsberg et al. [45] found signal activation in the PCC/ precuneus, TPJ, and middle and superior temporal gyrus during emotional-distancing tasks. Since the aMPFC contributes to the integration of emotion in decision-making and planning [46], activation in this area suggests that the stimuli may have elicited emotional processing. An attempt to relate the stimuli to the self also seems probable, due to activation of the precuneus, which has been shown to be involved in types of self-processing (e.g. mental imagery strategies; [47]). However, these strategies also engage precuneus perspective-based cognition. Perspective-based cognition has also been shown to involve the TPJ [48]. That both the precuneus and TPJ are involved in may suggest that subjects attempted to change their perspective when responding to the moral stimuli.

In the 3PP-based condition, subjects appear to evaluate the behavior of others through the inner characteristics of the actor, in accordance with the “actor-observer bias”. Behavioral data suggest that the evaluating standards were less strict, with 51% of the stimuli being rated as morally right. When compared to the non-moral condition neural activation during presentation of moral conditions was found in aMPFC, hippocampus (bilaterally), and visual cortex. That there was almost equal activation in the aMPFC for both 1PP- and 3PP presentations of moral conditions (as based upon ROI analysis) suggests the involvement of similar processes in these decision events. Activation in the visual cortex may be explained by the visual salience of the emotional stimuli presented. [28,49,50]. Due to dense interconnections between the visual cortex and the amygdala, a modulating effect from the amygdala as noted by previous studies seems possible [51].

Recent neuroimaging studies have related hippocampal activity to ToM in understanding the emotions and behaviors of others [39], specifically as related to the facilitative role of the hippocampus, and its implication in inducing and sustaining emotional reactions. Hippocampal activation may also suggest both a possible role of memories and projection of self-knowledge while making emotional judgments regarding others [40] and the viability of declarative memory to integrate relevant information between different inputs about a given event [52]. However, it has been suggested that ToM may be independent of episodic memory [53]. In the present study, the stimuli were not related to typical daily experiences, but rather, represented extreme violence, blasphemy, and questionable sexual behavior.

Therefore, we argue that activation in the 3PP condition may be dependent upon semantic memory, in that factual or general information about the world may contribute to making sense of perceived deviant behavior. Hippocampal activity has also been shown during tasks of semantic memory [54], in retrieval of relevant memories [55] that allow past events to influence present decisions [56]. Taking this into consideration, the presentation of moral situations may trigger the recollection of memories of related situational and/or contextual information that relates to, and could influence present decision-making through a Bayesian mechanism of ecological observation, orientation and action [2,5,34]. While it might be possible that the observed hippocampal activation could, perhaps partially, be explained by different conditions relying more or less on short-term memory, we find it difficult to explain why the 3PP would rely more on short-term memory than the 1PP, since there were no statistical significant differences in assessments of sentence length, valence, or arousal.

Furthermore, an interaction between the ventromedial prefrontal cortex (vmPFC) and hippocampus has been suggested to mediate cognitive evaluations of the moral character of others [57]. Emotional salience is attributed to moral information by the involvement of the vmPFC, while hippocampal networks involved in memory retrieval enable necessary contextual information in order to make an appropriate character judgment. However, given that the vmPFC includes at least the ventral part of Brodmann’s area 10 (BA 10; [58]), and appears to serve a binding function between aMPFC and the amygdala [59], we suggest that BA 10 may have a functional role in integrating emotional information (via enhanced activation of the visual cortex), and recollective aspects of the decision-process; (possibly through hippocampal connections) that are involved in, and/or subserve moral cognition and judgments.

Thus, we posit that the vmPFC plays a role in emotional salience, while the aMPFC contributes to synthesizing the “moral” information, by integrating emotional and recollective information, thereby enabling appropriate strategies in moral decision-making. To summarize, we claim that the involvement of the hippocampus for the 3PP moral judgment can be explained through the results of recent studies that elucidated its role in understanding emotions and behaviors of others, while somewhat more “classical” hippocampal activity (i.e.- memory) plays a role in the recollection of stored related retrograde situational or contextual information. We consider the role of the hippocampus in 3PP moral judgments of crucial importance due to the psychological implications of these functional roles.

There is also a temporal aspect that may be involved, which would support the “actor-observer bias”. If 1PP presentations engage evaluative cognition, then such processing is temporally related to the present [60,61]. The 3PP situation, however, relies on more abstract evaluations, which tend to be more time independent, in which inner characteristics of others may come into play. Moreover, if subjects distance themselves from the stimuli used in 1PP presentations, the time needed to evaluate these stimuli would be shorter than that needed to evaluate the stimuli in the 3PP condition, where memory processing would represent an important function in stimuli assessment.

An important aspect of the present study is the use of novel stimuli. Since moral dilemmas have already been used to study the "actor-observer bias" [27] a different approach, i.e. using moral reactions, may be helpful in extrapolating the findings. For this reason, control of emotional valence and duration of stimuli has been ensured. Such parameters, however, decrease the number of stimuli that were used. This may be problematic; however, due to the novelty of the approach used, a possible limitation in generalization seems suitable in order to gain greater experimental control over the stimuli.

Despite these limitations, the present findings suggest that different neural networks may be involved in, and subserve the perspective one has towards moral situations. A similar case was found for agency in moral judgments, for which different associated emotions were found to rely upon both distinct and overlapping neural substrates [62]. A psychological component, which could explain the neural differences found for moral perspective taking, is the actor-observer bias. Thus, care must be taken when interpreting neuroimaging studies of the neural bases of morality, since the perspective of the participants towards the moral stimuli may indeed elicit distinct neural activation.

In summary, moral stimuli presented in either 1- or 3PP elicit both distinct (e.g. hippocampus, and visual cortex for 3PP) and common patterns of neural activation (e.g. in the self- or ToM networks). These results suggest that differences may be related to the “actor-observer bias”. In the 1PP presentation the stimuli were evaluated with regard to the situation. Since the participants could not control the situation (although it elicited a strong emotional response), we posit that subjects may have attempted to distance themselves from the stimuli by engaging in perspective shifting. The 3PP moral judgments seem to have been evaluated by considering the inner characteristics of the “actors”, through recollection(s) of relevant information and also by engaging in ToM processes.

The overlap in the self- and ToM networks suggests that self-processing may be a basis through which to experience complex emotions about others' mental state [39]. These findings do not imply identical psychological processes for these different perspectives, and do not contradict the suggested involvement of the “actor-observer bias”. We believe that the most important implication of this study is related to distinct mechanisms and processes of moral cognition. To date, research has posed that networks of the so-called “moral brain” are homogenously activated, independent of the eliciting stimuli. This also implies that similar psychological processes subserve moral cognition and/or reasoning, irrespective of perception of, or orientation to the situation [15]. The present results, however, contrast this view, and suggest that different types of stimuli may indeed engage distinct types of neural activity and psychological processing, and that both reflect orientation to the situation, which may be influenced by a host of factors affecting cognitive biasing, inclusive of cultural differences and a variety of social effects.

While it has been offered that moral and ethical judgments and actions are “other-based” (see, for example, MacMurray [63]), it is important to note that any and all decisions - inclusive of moral judgments (affecting others) - emanate from, and in many ways are reciprocal to, and reflective of the self [2,3,5,64-66]. In this light, potentially moral situations are perceived differently depending upon one’s orientation to, and relative involvement in the situation and circumstance, and its effect upon prior experience, past and present reinforcing and rewarding influences, and predicted outcomes and their impact upon self and others [67-69].

The results presented here suggest that while there appears to be something of a core neural network that is involved in the types of moral decisions rendered in this study, the spatial and temporal engagement of elements of this network are peculiar to distinct types and aspects of situation and circumstances. There are several limitations of this study. First, the number of stimuli remaining after the pilot study was rather small. Therefore, we suggest that future studies employ a larger number of stimuli. This would also enable non-repetition of stimuli, thereby avoiding possible learning effects that have been shown to decrease BOLD signal – e.g. in visual cortex, PFC etc. [69,70]. Second, it remains somewhat uncertain to what extent participants attributed external causes to the 1PP, and internal causes to the 3PP, since the subjects were not required to describe the way in which they evaluated the stimuli. Future studies could employ a post-scanning interview during which subjects are asked to explain their decision-making processes.

## Conclusion

In conclusion, we opine that the present study suggests differential patterns and mechanisms of 1PP and 3PP moral judgments. Such findings have implications for consideration of how moral decisions are made and morally-relevant acts are tendered (e.g.- “Good Samaritan” acts, “by-stander effects”, etc.), and prompt further inquiry to how patterns of neural activity may affect types and extent of behaviors in morally-relevant situations, and if and how such patterns of activity are stable, modifiable, and/or learned. Yet, we also advocate prudence in interpretation of these and related findings [2-4,7-9], as the limitations of fMRI, like any neurotechnology, must be appreciated (see van Meter [71] for overview).

This encourages engagement of neuroethics’ second tradition, namely, an analysis of the ways that neuroscience and neurotechnology are, can, and should be employed to gain understanding of cognitions, emotions and behaviors, and how such information is used (in medicine, law and the public sphere). Indeed, while findings such as those presented in this study may be provocative, care must be taken in extrapolating such information to real-world circumstances, so as to avoid over- or under-estimating the role of neurobiology in psychological and social activity, and/or the capabilities of neuroscience and neurotechnology to address and answer perdurable and pressing questions about the “nature” of morality, and other dimensions of cognition, emotion and behavior.

## Competing interests

The authors declare that they have no competing interests.

## Authors’ contributions

All authors contributed to study concept. MA was responsible for data collection, data analysis and interpretation, and manuscript preparation. EG and KF were responsible for data analysis and interpretation and critical review of the manuscript, MR and JB were responsible for data collection and preliminary data evaluation. YB, EP and JG made substantial contributions to interpretation of data, have been involved in developing and revising the manuscript for important intellectual content, and have given final approval of the version to be published.
